# The therapeutic link in the speech therapy clinic: a necessary reflection

**DOI:** 10.1590/2317-1782/20232022167en

**Published:** 2023-10-09

**Authors:** Adriele Barbosa Paisca, Lucas Jampersa, Rosane Sampaio Santos, Roxele Ribeiro Lima, Maria Regina Franke Serratto, Cristiano Miranda de Araújo, Giselle Aparecida de Athayde Massi

**Affiliations:** 1 Programa de Mestrado e Doutorado em Distúrbios da Comunicação, Universidade Tuiuti do Paraná - UTP - Curitiba (PR), Brasil.; 2 Associação Educacional Luterana BOM JESUS/IELUSC - Joinville (SC), Brasil.; 3 Coordenação da Graduação de Fonoaudiologia, Universidade Tuiuti do Paraná - UTP - Curitiba (PR), Brasil.

**Keywords:** Therapeutic Bond, Therapeutic Relationship, Speech Therapy, Clinic

## Abstract

**Purpose:**

To understand the meanings that the therapeutic bond assumes for clinical speech therapists.

**Methods:**

The research was approved by the Ethics Committee, being of a transversal character, with a quantitative-qualitative approach in the Content Analysis. The research with the participation of 96 clinical speech therapists, registered in the Speech Therapy Council of the 3rd region (CRFa 3), which covers the States of Paraná and Santa Catarina.

**Results:**

Of the 96 speech therapists included, a significant part of the participants defined the therapeutic bond as a relationship/interaction. Regarding the role of the bond for the speech therapy clinical work, most professionals declared theirs as a fundamental basis and another part of the bond is necessary for the evolution/development of the patient.

**Conclusion:**

It is possible to understand that, according to the therapeutic patients, it is essential to sustain, maintain the clinical work for users, impacting the resignification of the complaint and the minimization of the users' suffering.

## INTRODUCTION

The therapeutic bond depends on the relationship between clinicians and subjects who seek them in suffering. Such a relationship involves feelings of trust, security, care, or their opposites, distrust, hostility, and opposition, depending on the previous histories of the people involved^(1)^. The alliance established between clinical speech-language pathologists and patients directly influences the development of the therapeutic process, including the mitigation of suffering given the complaints and symptoms the patient presents^(2)^.

However, studying this relationship is a challenging exercise. It involves a search to understand the complexity of human relationships, which undergo successive adaptations and transformations, influenced by the internal and external interactions of subjects with themselves and others^(3)^. Thus, to scrutinize the therapeutic bond, we must distinguish the theoretical bases and aspects defining it.

According to a psychoanalytic perspective, there are basically two types of bonds: positive and negative. The first relates to a feeling close to love, and the second is better known as hostility. Therefore, the bond should not be considered only as the transfer of feelings of affection or empathy. Rather, it also includes feelings of anger and hatred and sometimes expresses indifference^(4)^.

These feelings manifested in the clinic indicate the possibilities and difficulties faced by patients and clinicians when establishing the therapeutic bond. However, regardless of the affection patients direct to therapists and vice-versa, it is important to clarify that both feelings stem from unconscious fantasies associated with the first bonds established in the lives of each person^(5)^.

According to the psychological theory of attachment, which maintains intersections with the psychoanalytic perspective, the relationship between the baby and the mother or other people caring for the child is decisive for the child's development and developing future relationships^(6)^. The care given to babies, more or less affectionate, leads children to delimit an internal representational model of themselves. This self-image, elaborated according to the exchanges between them and their caregivers, explains a bonding pattern the subject tends to resume in other significant interpersonal relationships, including with professionals they may choose for clinical work^(3)^.

According to Collective Health, the bond is considered a conditioner of health processes insofar as it assumes the role of enabling co-responsibility, continuity, and longitudinally of care. The alliances bonding health workers and their patients must be based on relationships of affection and trust, enabling a deepening of co-responsibility for health, assuming a therapeutic potential^(7)^.

Thus, considering the different perspectives aimed at explaining the bond and, regardless of the theoretical position adopted, there is a remarkable agreement that the patient-professional relationship plays a relevant role in health actions. It is understood that speech-language pathology clinical practices should value this relationship. When considering the bond, therapists can broaden and qualify their listening, welcoming the uniqueness of the story narrated by the patient^(1)^.

From a clinical perspective, a crucial aspect that must be considered when establishing the therapeutic bond is the posture assumed by the speech-language pathologist when faced with the patient's complaint, including the development of a work capable of taking a clinical stance based on a supposed knowledge^(5)^. This indispensable attitude in the therapeutic process requires therapists to understand their roles and limits so that the therapeutic environment does not become a place of power over the other. However, in the opposite direction, such a space should provide the establishment of a horizontal relationship, from which patients can feel welcomed and strengthened to explain their singularities^(5)^.

The change in the professional's gaze away from pathology to focus on the suffering subject enables an approach that opens up space to resignify the patient's symptom^(8)^. Some studies state that the therapeutic bond is essential for building and sustaining the therapeutic process because it brings the professional closer to the subject's truths, improving their adherence and engagement in this process^(2-11)^.

However, even though speech-language pathologists consider that, in general, the bond is essential for the referral of clinical work, there is a limited number of studies in speech-language pathology seeking to deepen this theme^(12)^. Nevertheless, it is worth noting that a superficial understanding of the bonding phenomenon tends to lead professionals to reproduce a practice based on re-educational techniques without considering the subjects and their singular histories^(1)^. In this direction, the speech-language pathology clinic seeks the normalization and standardization of subjects. It uses procedures that make it impossible to establish a therapeutic relationship through which patients can accept and resignify their symptoms, mitigating their suffering^(4)^. Thus, based on this understanding, this study aimed to understand the meanings the therapeutic bond assumes for clinical speech-language pathologists.

## MATERIALS AND METHODS

This is a cross-sectional study with a quantitative and qualitative approach. It is based on Content Analysis (CA), which aims to analyze linguistic materials. The Ethics Committee approved it with the document no. 34894720.6.0000.8040. All research participants signed the Informed Consent Form (ICF).

### Material

In order to carry out this investigation, we prepared a semi-structured electronic questionnaire and implemented it on the Google Forms platform. The questionnaire (Appendix A) comprised 23 questions (11 closed and 12 open). The closed questions were designed to obtain socio-demographic information from the professionals, such as the participants' ages, state of residence (Paraná or Santa Catarina), training time, academic level, clinical practice time, area of activity, the age group for whom they provide care, and whether they were professionally active during this study. The open questions aimed to direct the understanding of the therapeutic bond's implication in speech-language pathology clinical practice from the perspective of speech-language pathologists.

In order to verify the tangibility of the questionnaire before sending the instrument to all professionals registered with CRFa 3, we conducted a pilot study with 12 speech therapists living in Paraná and Santa Catarina, captured by the snowball technique. This qualitative technique proposes that, after locating some people who match the study profile, they are invited to recommend other subjects capable of integrating the research and so on, progressively^(13)^. These 12 professionals responded, evaluated, and suggested improvements to the data collection instrument, enabling the authors to observe gaps in it. These gaps were revised and adapted, enabling the instrument's application to more participants.

### Participants

The study included 146 clinical speech-language pathologists with active registration in the Speech-Language Pathology Council of the 3rd region (CRFa 3), which covers two southern Brazilian states (Paraná and Santa Catarina). Data collection was organized based on the inclusion of answers from 96 speech-language pathologists who met the eligibility criteria. This study preserved, coded, and recognized the participants' identities using Arabic numbers ranging from 1 to 96.

Inclusion criteria included professionals with a clinical practice of one year or more. On the other hand, exclusion criteria included professionals not working in the speech-language pathology clinic at the time of data collection.

### Data collection procedures

The electronic data collection instrument was sent through the Speech-Language Pathology Council of the 3rd Region (CRFa 3), by e-mail, to 4,297 professional speech-language pathologists registered with the organization. Along with the electronic questionnaire, we sent the Informed Consent Form (ICF) and a summary of the project, which explained its objective and justification. On March 13, 2021, we sent the questionnaire to professionals. However, due to the low number of answers, we sent the questionnaire again on April 26, 2021, through the CRFa 3. Therefore, the study included all professionals who were willing to answer the questionnaire and who met the inclusion criteria within two months after the instrument was first sent.

### Data analysis

Data analysis was based on Content Analysis (CA). Content Analysis has a quantitative-qualitative character and allows using statistical parameters to study communication phenomena^(14)^. It is an analysis method that considers objective and subjective aspects of texts produced during the research to discuss the data collected through systematic procedures that can organize quantitative and qualitative indicators^(15,16)^. Specifically, CA is defined as a set of methodological tools, which analyzes different verbal and non-verbal content sources in a refined and critical way, increasing its level of effectiveness^(16)^.

#### Quantitative analysis

The quantitative analysis sought to characterize the socio-demographic profile of the participating speech-language pathologists through descriptive statistical analysis. We used the chi-square test to verify the existence of an association between the different categorical variables evaluated. All analyses were performed in the statistical software Jasp, version 0.14.1, with a 5% significance level.

#### Qualitative analysis

Regarding the linguistic and discursive materials collected through the questionnaire, which sought to apprehend what speech-language pathologists understand about the therapeutic bond in Speech-Language Pathology, we organized them following the CA procedures^(14)^. In this regard, we conducted thematic and lexical analyses to organize this study's categories.

The organization followed the three phases of CA: 1) Pre-analysis, in which the material obtained in the data collection is prepared; 2) Exploration of the material, stage in which the categories are grouped based on the registration units, according to their common characteristics, around the words and themes mentioned by the participants; 3) Treatment of Results, phase in which the interpretation of the research data is performed, making it possible to produce inferences^(17)^.

## RESULTS

Table 1 shows the profile of the 96 professionals involved considering their sociodemographic aspects.

**Table 1:** Characteristics of the study population

*Variable*		*n*	*%*
Training time			
	*3-7 years*	26	21.1
	*8-14 years*	*19*	*19.8*
	*15-19 years*	*15*	*15.6*
	*Over 20 years*	*36*	*37.5*
Academic level			
	*Undergraduate degree*	18	18.8
	*Specialization/Improvement*	58	60.4
	*Masters degree*	16	16.7
	*Doctorate degree*	4	4.2
Time working in clinical therapy			
	*1-4 years*	15	15.6
	*5-9 years*	*23*	*24*
	*10-19 years*	*25*	*26*
	*Over 20 years*	*33*	*34.4*
Area of activity*			
	*Dysphagia/Motricity*	*66*	*68.8*
	*Language*	*82*	*85.4*
	*Voice*	*27*	*28.1*
	*Audiology*	*17*	*17.7*
Search for some specific training on the therapeutic bond.			
	*Yes*	*29*	*30.2*
	*No*	*67*	*69.8*
Type of training specifically aimed at broadening the therapeutic bond understanding. *			
	*Courses*	*22*	*75.9*
	*Seminars*	*5*	*17.2*
	*Scientific congresses*	*2*	*6.9*
	*Lectures*	*15*	*51.7*
	*Study groups*	*16*	*55.2*
	*Others*	*7*	*24.1*
Age (years) - Mean (SD)		40.5 (11.2)	

* There was a possibility for the professional to select more than one answer to this question

Source: The authors

A total of 100% of the 96 speech-language pathologists included were professionally active and working as clinical speech-language pathologists. Regarding the length of training, 37.5% were speech-language pathologists who had graduated for more than 20 years, followed by professionals with three to seven years of training, totaling 21.1%. Regarding the academic level, there was a predominance of 60.4% of participants with specialization/improvement, followed by 18.8% who had only undergraduate degrees, and only 4.2% had a doctorate degree.

From the total number of professionals who participated in this study, 34.4% had been working clinically for more than 20 years, and 15.6% between one and four years. Regarding the area of activity, there is a greater number of participating speech-language pathologists who worked with language (85.4%), followed by those working with dysphagia/motricity (68.8%). Professionals working in the field of voice accounted for 28.1%, and those working in audiology, 17.7%.

Regarding complementary training specifically focused on the therapeutic bond, only 30.2% sought further training on the subject. When this 30.2% was asked about the type of training they had, 72.9% stated that they had attended courses, 55.2% participated in study groups, 51.7% in lectures, 17.2% in seminars, 6.9% in scientific congresses, and 24.1% sought other means to deepen their knowledge on therapeutic bond.

### Axis 1 - Representation of the therapeutic bond

Table 2 seeks to clarify the research participants' understanding of the therapeutic bond in the speech-language pathology clinical context. Thus, participants answered what bonding is from their perspective. The answers given by the speech-language pathologists were organized into the following categories: relationship/interaction, trust/acceptance, listening/empathy, and others.

**Table 2:** Characterization of the answers obtained on therapeutic bond

**AXIS 1 - REPRESENTATION OF THE THERAPEUTIC BOND**			
Explanation of what is the therapeutic bond according to speech-language pathologists			
Categories	*n*	*%*	Examples of recording units
*Relationship/Interaction*	46	47.9	The close relationship/interaction between patient/therapist (1).
			Interaction between professional, patient, and family (43).
*Trust/Acceptance*	*25*	26	Family trust in the therapist's decisions (22).
			The therapeutic bond involves trust between the patient and therapist and welcoming the patient (8).
*Listening/Empathy*	*2*	2.1	A process that takes place through qualified and active listening by the therapist (14).
			It has to do with empathy (47).
*Others*	*15*	15.6	Associate care with a multidisciplinary team (10).
			Include the family in the therapeutic process (60).
*No answer*	*8*	8.3	

Source: The authors

In axis 1, most professionals defined the therapeutic bond as a relationship/interaction (47.9%). Another 26% defined it as trust/acceptance. However, it is worth noting that 26% did not answer the question.

### Axis 2 - The role of the therapeutic bond according to speech-language pathologists

In order to organize this axis, the professionals were asked about the role of the therapeutic bond in the speech-language pathology clinical practice. The answers were grouped into categories related to affinity/trust, base/fundamental, motivation/engagement, evolution/development, and others, as shown in Table 3.

**Table 3:** Characterization of the answers obtained on the therapeutic bond's role

**AXIS 2 - THE ROLE OF THE THERAPEUTIC BOND ACCORDING TO SPEECH-LANGUAGE PATHOLOGISTS**			
Categories	*n*	*%*	Examples of recording units
*Affinity/Trust*	12	12.5	Therapeutic work can only be carried out through the trust established in the bond (50).
			Without the bond, there is no exchange in the relationship and no trust from the patient and/or family member (9).
*Base/Fundamental*	37	38.5	Fundamental role since, without a bond, we will hardly achieve our goals (17).
			It is one of the necessary cornerstones for therapeutic success (21).
*Motivation/Engagement*	8	8.3	It is what motivates (4).
			It is the first step in facilitating therapeutic engagement (55).
*Evolution/Development*	24	25	Expand possibilities for patient development (7).
			It is necessary for patient evolution (45).
*Others*	*12*	12.5	No therapeutic bond cancels treatment (63).
			Strengthens planned strategies (87).
*No answer*	*3*	3.1	

Source: The authors

Regarding the therapeutic bond's role in clinical speech-language pathology work, the professionals most significantly described it as the basis/fundamental (38.5%) and necessary for the patient's evolution/development (25%). Furthermore, 8.3% related the bond to motivation/engagement in therapeutic work, 12.5% described other characteristics, and 3.1% did not answer the question.

### Quantitative results

**Figure 1:**
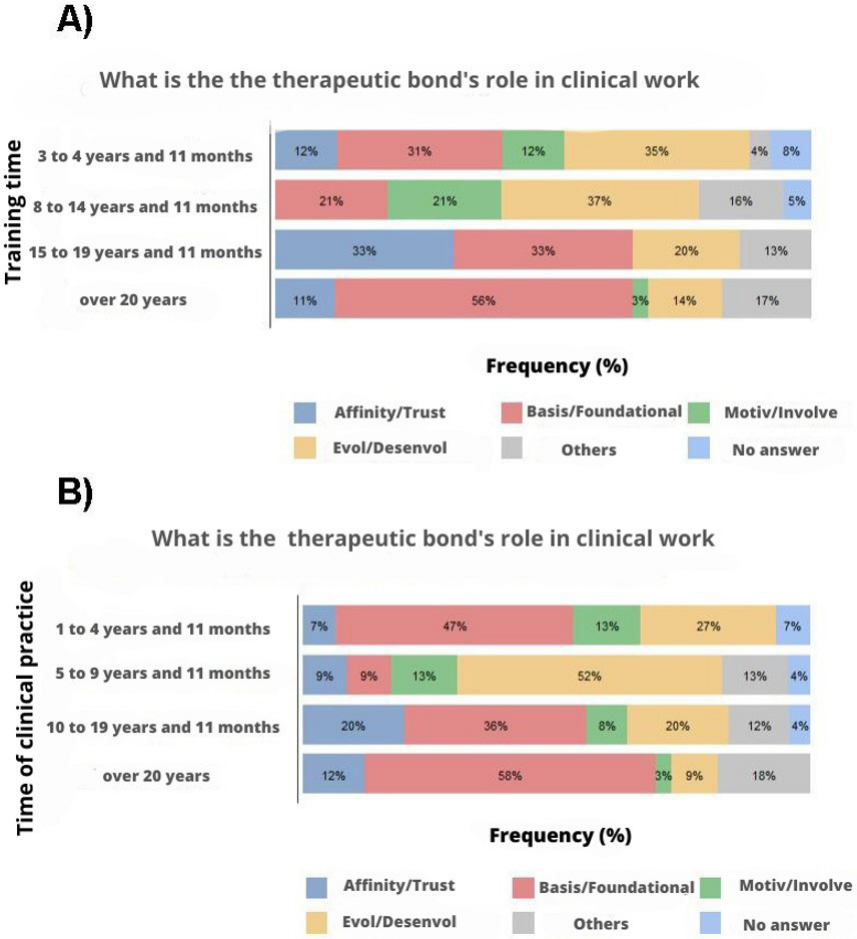
Graphical demonstration of the results of the information cross-checks that showed significance

There was an association between the participants' training time and their answers regarding the therapeutic bond's role (p = 0.036). For professionals who graduated more than 20 years ago, the bond is the foundation of the speech-language pathology clinic. Meanwhile, for professionals who graduated less than 14 years ago and more than eight years ago, the bond is responsible for the evolution and development of the therapeutic work (Figure 1A).

Caption: Chi-square test; 5% significance level

There was also an association between the time of clinical practice and the participants' answers regarding the therapeutic bond's role (p= 0.050). For speech-language pathologists who have been working clinically for more than 20 years, the therapeutic bond was the basis of their work. Meanwhile, for professionals who have been working for more than five and less than ten years, the bond is an element that develops the therapeutic work (Figure 1B).

## DISCUSSION

The results presented in Table 1 regarding the participants' characterization show that a significant portion of the professionals has been trained and working clinically as speech-language pathologists for more than 20 years. These professionals are more interested in collaborating with this study, which focuses on the therapeutic bond in the speech-language pathology clinic. This data indicates that the bond's role tends to become more significant as professionals have more time in training and, therefore, in clinical practice.

Regarding the participants' area of activity, the predominance of professionals working in the language area is noteworthy. The predominance of speech-language pathologists who work clinically in language can be justified because language is one of the essential cornerstones for establishing and sustaining the bond. For it is through language that every human being constitutes itself as a subject, understands the world, and acts upon it^(18)^. The therapeutic bond allows access through language to the conscious and unconscious contents of individuals, bringing out their subjectivity, so necessary for the referral of speech-language pathology clinical work^(19)^.

Moreover, the therapeutic bond is associated with significant improvements in the communication skills of subjects undergoing speech-language pathology treatment^(2)^. The therapeutic relationship should also be considered in rehabilitating subjects with chronic orofacial pain. According to the author, such a relationship broadens the therapist's listening, making it possible to assist subjects in relieving their pain, to the extent that their life story is considered in the understanding of their symptoms^(4)^.

It is worth mentioning that even though the audiology literature recognizes the importance of the bond between the speech-language pathologist and the patient in the audiological clinical work, the number of audiologists who agreed to participate in this study was limited. According to a study focused on the audiological rehabilitation of older people, the therapeutic bond is considered fundamental, as it impacts the reduction of postponement of consultations and adherence to the proposed treatment^(20)^. In this same study, the bond is also associated with the fact that patients feel valued and listened to by the professional and not just conceived as consumers and buyers of hearing aids^(20)^.

Another point worth noting in the results shown in Table 1 is that almost 70% of the professionals did not undergo specific training on topics involving the therapeutic bond so that they could base their clinical practice. Therefore, even though the professionals point out the therapeutic bond as an essential prerequisite for their work's referral, they are not being trained to meet this fundamental prerequisite related to their work. It is important to reflect on the reasons that explain this situation, which may be related to a lack of interest or, more than that, the lack of training actions to meet this demand.

### Axis 1 - Representation of the therapeutic bond

Axis 1, which, according to Table 2, explains what speech-language pathologists understand by therapeutic bond, indicates a significant predominance of professionals who understand it as the relationship established between the therapeutic process participants (the speech-language pathologist and the patient). This relationship is the guiding line responsible for forwarding the speech-language pathology work. In this regard, according to psychoanalytic studies, it is necessary to understand that the therapeutic bond is a dynamic activity in constant movement, which includes both the subjects involved and their previous histories^(21)^.

Therefore, the bonding relationship, constituted in the therapeutic sphere, is permeated by the coexistence of the parallel between the affective experiences lived by the subjects throughout their history and the relational experiences elaborated in the therapeutic space, as proposed by the attachment theory^(6)^. Furthermore, there is a need for patients to be considered and understood as biopsychosocial beings. Thus, it is possible to resignify the anguish and the symptom they experience. Such achievement is only possible through the relationship established between patients and therapists, which allows professionals to recognize the need of the subjects who seek them and thus intervene more effectively, helping them in mitigating their suffering^(22,23)^.

In this perspective, following the first sub-axis, another part of the research participants related the therapeutic bond to listening, trust, and acceptance, among others. Qualified therapeutic listening promotes holistic care, which can strengthen bonds, enabling an approximation between the professional and the truths reliably related to the subject's real suffering^(24)^. According to Collective Health studies, qualified listening maximizes the therapeutic potential. It contributes to consolidating the therapeutic relationship, as it respects the patient's uniqueness, strengthening the trust and participation of those involved in the therapeutic process^(7)^.

Regarding trust, it should be considered one of the primary points of therapeutic work, as it allows patients to feel comfortable and safe to share their life stories, promoting more authentic interactions toward the re-elaboration of the symptom. However, such trust only becomes possible based on welcoming the patient, making them feel safe to bring out their uniqueness^(25)^.

### Axis 2 - The role of the therapeutic bond according to speech-language pathologists

Regarding the therapeutic bond's role in speech-language pathology clinical practice, 38.5% of professionals understand it as a fundamental basis for their clinical practice. The therapeutic bond is pointed out as paramount for designing the therapeutic work because, in addition to building a secure base that helps patients address their demands, the relationship between patients and clinicians can contribute to subjects suffering and can resignify their previous affective bonds. According to the attachment theory, especially in circumstances in which the subjects grew up in interpersonal contexts based on distrust and fear, the therapist can assume a position that can promote a safe bond. This safe bond leads the patient to face another relationship model supported by a link that enables trust and security, reworking difficult relationships experienced in previous situations^(3)^.

Furthermore, from a psychoanalytic perspective, the therapeutic bond should be considered an underlying factor in all clinical practice. Therefore, it is a necessary condition for therapeutic work referral. In other words, it is an element that permeates all clinical activity, without which, tendentiously, there is no possibility of treatment, care, and/or resignification of the symptom^(19)^.

Motivation and engagement were associated with the therapeutic bond's role by 8.3% of the speech-language pathologists participating in this study. According to a study based on Collective Health, the therapeutic relationship, insofar as it opens space for subjects to explain their particularities, allows them to understand their role in the work to be developed, leading them to assume an active position in the relationship with the clinician^(26)^. In this direction, the therapeutic bond can significantly improve the engagement, motivation, autonomy, and social participation of subjects inside and outside the therapeutic setting^(9)^.

A significant proportion of 25% of the speech-language pathologists related the bond to the therapy's evolution and development. Regarding this relationship, it is worth noting that the therapeutic bond should not be associated only with “cure” or symptom suppression but as an opportunity that allows patients to reframe their life stories, pains, and previous bonds. According to the National Health Promotion Policy, health care should not be limited only to disease recovery but to an activity in which the professional, through qualified and welcoming listening, promotes the maximization of autonomy, empowerment, and social participation of patients, assisting them in the process of becoming the author of their history actively and consciously^(27)^.

## FINAL CONSIDERATIONS

Regarding the understanding of speech-language pathologists about the meanings that the therapeutic bond assumes in the clinical practice of speech-language pathology, it was postulated as an essential part of the design and outcome of the therapeutic process. It can strengthen motivation and engagement factors and help subjects resignify their symptoms.

## Appendix A Structured questionnaire for data collection.

**Table:**

CHARACTERIZATION DATA:
1. Age: _________
2. State of residence:
() Paraná () Santa Catarina
3. Works in:
() Capital () Metropolitan area () Countryside
4. Time of undergraduate training:
a) () 1 to 2 years and 11 months
b) () 3 to 7 years and 11 months
c) () 8 years to 14 years and 11 months
d) () 15 years to 19 years and 11 months
e) () over 20 years
5. Institution where you graduated?
6. Academic level:
a) () undergraduate degree
b) () Specialization In which area?
c) () Masters degree In which area?
d) () Doctorate degree In which area?
7. Are you currently professionally active?
a) () Yes
b) () No
8. Area(s) of activity: * Please note that there may be more than one answer.
a) () Language, for how long?
b) () Dysphagia, for how long?
c) () Motricity, for how long?
d) () Educational Speech-Language Pathology, for how long?
e) () Voice, for how long?
f) () Gerontology, for how long?
g)() Audiology, for how long?
h)() Neurofunctional Speech-Language Pathology, for how long?
i) () Collective Health, for how long?
j) () Occupational Speech-Language Pathology, for how long?
h)() Neuropsychology, for how long?
l) () Fluency, for how long?
9. What is your field of expertise: * Please note that there may be more than one answer
a) () Clinic
b) () Hospitals/Maternities
c) () Home Care
d) () Health facility
e) () Higher education institutions
f) () Companies/Industries
g)() Regular/Special schools
h)() Others, Which?
10. How long have you been providing therapeutic clinical care?
a) () less than one year
b) () 1 to 4 years and 11 months
c) () 5 years to 9 years and 11 months
d) () 10 years to 19 years and 11 months
e) () over 20 years
11. What age group(s) do you provide care for? * Please note that there may be more than one answer.
a) () Babies - from 0 to 1 year old
b) () Children - from 2 to 11 years
c) () Adolescents - 12 to 18 years old
d) () Adults
e) () Older people
Issues related to the therapeutic bond in speech-language pathology clinical practice:
12. What is your understanding of the therapeutic bond?
13. If you could spell out therapeutic bonding in one word, which word would you use?
14. Do you seek to establish a therapeutic bond with the people you see clinically?
a) () Yes
b) () No
Justification:
15. How do you realize that the link has been established?
16. What is the relevance of the bond during the therapeutic process?
17. What is the speech-language pathologist's role in establishing the therapeutic bond?
18. Have you ever faced cases where it was difficult to establish a therapeutic bond?
a) () Yes
b) () No
If so, in your opinion, what factors were involved in this difficulty?
19. If, in the clinic, you seek to establish a bond, is this establishment based on any theoretical approach?
a) () Yes
b) () No
If yes, which one?
20. Have you sought any more specific training on therapeutic bonding?
a) () Yes
b) () No
If yes, what kind of training?
a) () courses
b) () lectures
c) () seminars
d) () scientific congresses
e) () study groups
f) () others: which ones ________________________________________________
21. Any other remarks/comments/statements you wish to make:
