# Supplementary material for: The therapeutic link in the speech therapy clinic: a necessary reflection
Source: Codas. 2023 Oct 9;35(6):e20220167. doi: 10.1590/2317-1782/20232022167en (PMC10702705; doi:10.1590/2317-1782/20232022167en)
Supplement: Translation

# Translation: O vínculo terapêutico na clínica fonoaudiológica: uma reflexão necessária

**DOI:** 10.1590/2317-1782/20232022167pt

**Authors:** Adriele Barbosa Paisca, Lucas Jampersa, Rosane Sampaio Santos, Roxele Ribeiro Lima, Maria Regina Franke Serratto, Cristiano Miranda de Araújo, Giselle Aparecida de Athayde Massi

**Affiliations:** 1 Programa de Mestrado e Doutorado em Distúrbios da Comunicação, Universidade Tuiuti do Paraná - UTP - Curitiba (PR), Brasil.; 2 Associação Educacional Luterana BOM JESUS/IELUSC - Joinville (SC), Brasil.; 3 Coordenação da Graduação de Fonoaudiologia, Universidade Tuiuti do Paraná - UTP - Curitiba (PR), Brasil.

**Keywords:** Vínculo Terapêutico, Relação Terapêutica, Fonoaudiologia, Clínica

## Abstract

**Objetivo:**

Compreender os sentidos que o vínculo terapêutico assume para fonoaudiólogos clínicos.

**Método:**

A pesquisa foi aprovada por Comitê de ética, sendo de caráter transversal, de abordagem quanti-qualitativa, pautada na Análise do Conteúdo. A pesquisa contou com a participação de 96 profissionais fonoaudiólogos clínicos, inscritos no Conselho de Fonoaudiologia da 3ª região (CRFa 3), o qual abrange os Estados do Paraná e de Santa Catarina.

**Resultados:**

Dos 96 fonoaudiólogos incluídos, parte significativa dos participantes definiram o vínculo terapêutico como sendo relação/interação. Referente ao papel do vínculo para o trabalho clínico fonoaudiológico, a maioria dos profissionais o descreveu como base fundamental e outra parte deles afirmou que tal vínculo é necessário para a evolução/desenvolvimento do paciente.

**Conclusão:**

É possível compreender que, de acordo com os participantes, a relação terapêutica é essencial para a sustentação e manutenção do trabalho clínico fonoaudiológico, impactando na ressignificação da queixa e na minimização do sofrimento dos usuários.

## INTRODUÇÃO

O vínculo terapêutico depende da relação constituída entre um profissional clínico e o sujeito que o busca em sofrimento, sendo que tal relação envolve sentimentos de confiança, segurança, cuidado, ou os seus opostos, desconfiança, hostilidade, oposição, a depender das histórias pregressas das pessoas envolvidas^(1)^. A aliança firmada entre o fonoaudiólogo clínico e o paciente tem influência direta no desenvolvimento do processo terapêutico, incluindo a minimização do sofrimento diante das queixas e sintomas que tal paciente apresenta^(2)^.

Contudo, estudar essa relação é um exercício desafiador, pois envolve uma busca por compreender a complexidade das relações humanas, que sofrem sucessivas adaptações e transformações, influenciadas pelas interações internas e externas do sujeito, consigo mesmo e com os outros^(3)^. Dessa forma, para perscrutar o vínculo terapêutico é preciso distinguir bases teóricas e aspectos que o definem.

De acordo com uma perspectiva psicanalítica, basicamente, existem dois tipos de vínculo: o positivo e o negativo. O primeiro está ligado a um sentimento que é próximo ao amor, e o segundo é mais conhecido como hostilidade. Por isso, o vínculo não deve ser considerado apenas como a transferência de sentimentos de carinho, afeto ou empatia. Ao contrário, ele, também, abarca sentimentos de raiva e de ódio, além de explicitar, por vezes, indiferença^(4)^.

Esses sentimentos, manifestados na clínica, são indicativos de possibilidades e de dificuldades enfrentadas pelo paciente e pelo clínico, na constituição do vínculo terapêutico. Mas, independentemente dos afetos que o paciente direciona ao terapeuta e vice-versa, cabe esclarecer que os sentimentos de ambos decorrem de fantasias inconscientes, associadas aos primeiros vínculos estabelecidos, na vida de cada um^(5)^.

Conforme a teoria psicológica do apego, que mantém pontos de intersecção com a ótica psicanalítica, a relação que se organiza entre o bebê e a mãe ou outras pessoas que lhe dedicam cuidados, em seus primeiros anos de vida, é determinante para o desenvolvimento da criança e para a construção de suas relações futuras^(6)^. Os cuidados destinados ao bebê, mais ou menos afetivos, levam a criança a delimitar um modelo representacional interno de si mesma. Essa autoimagem, elaborada em função das trocas que se dão com os seus cuidadores, explicita um padrão de vínculo que o sujeito tende a retomar em outras relações interpessoais significativas, inclusive, com profissionais que ele venha a eleger para um trabalho clínico^(3)^.

Na concepção da Saúde Coletiva, o vínculo é visto como condicionante dos processos de saúde, na medida em que assume o papel de viabilizador da corresponsabilidade, continuidade e longitudinalidade do cuidado. As alianças que vinculam trabalhadores de saúde e seus usuários devem se embasar em relações de afeto e confiança, capazes de permitir aprofundamento de corresponsabilização pela saúde, assumindo um potencial terapêutico^(7)^.

Assim, tendo em vista a existência de diferentes perspectivas que se propõem a explicar o vínculo e considerando que, independentemente do posicionamento teórico adotado, há uma notável concordância de que a relação paciente-profissional desempenha relevante função nas ações voltadas à saúde, entende-se que as práticas clínicas fonoaudiológicas devem valorizar essa relação. Pois, ao considerar o vínculo, o terapeuta pode ampliar e qualificar a sua escuta, acolhendo a singularidade da história narrada pelo paciente^(1)^.

De um ponto de vista clínico, um aspecto crucial a ser considerado, na constituição do vínculo terapêutico, é a postura assumida pelo fonoaudiólogo, frente a queixa do paciente, incluindo o desenvolvimento de um trabalho capaz de assumir uma postura clínica fundamentada em um suposto saber^(5)^. Essa postura, indispensável no processo terapêutico, exige do terapeuta a compreensão do seu papel e de seus limites, para que o ambiente terapêutico não se torne um lugar de poder sobre o outro. Mas, em direção oposta, que tal espaço proporcione o estabelecimento de uma relação horizontalizada, a partir da qual o paciente possa se sentir acolhido e fortalecido para explicitar a sua própria singularidade^(5)^.

A mudança de olhar do profissional esvaindo-se da patologia, para focalizar o sujeito em sofrimento, viabiliza uma conduta que abre espaço para ressignificar o sintoma do paciente^(8)^. Alguns estudos afirmam que o vínculo terapêutico é essencial para a construção e para a sustentação do processo terapêutico, pois, aproxima o profissional das verdades do sujeito, potencializando a sua adesão e o seu engajamento nesse processo^(2-11)^.

Entretanto, apesar de os fonoaudiólogos considerarem que, de forma geral, o vínculo é essencial para o encaminhamento de um trabalho clínico, há um número restrito de pesquisas, no campo da Fonoaudiologia, que buscam aprofundamento sobre essa temática^(12)^. Ressalta-se, porém, que um entendimento superficial sobre o fenômeno vincular, tendenciosamente, leva o profissional a reproduzir uma prática baseada em técnicas reeducativas, sem levar em conta o sujeito e sua história singular^(1)^. Nessa direção, a clínica fonoaudiológica acaba por buscar a normalização e a padronização dos sujeitos, utilizando-se de procedimentos que impossibilitam a constituição de uma relação terapêutica, por meio da qual o paciente pode aceitar e ressignificar seus sintomas, mitigando o seu sofrimento^(4)^. Assim, a partir desse entendimento, a presente pesquisa objetiva compreender os sentidos que o vínculo terapêutico assume para fonoaudiólogos clínicos.

## MATERIAIS E MÉTODOS

Esse estudo é de caráter transversal e de abordagem quanti-qualitativa, pautada na Análise do Conteúdo (AC), a qual se propõe a analisar materiais linguísticos. Foi aprovado pelo por Comitê de Ética, com documento nº. 34894720.6.0000.8040. E todos os participantes da pesquisa assinaram o Termo de Consentimento Livre e Esclarecido (TCL).

### Material

Para a realização da presente investigação, foi elaborado um questionário, eletrônico semi-estruturado, sendo que o mesmo foi implementado na plataforma *Googles forms*, visando atender ao objetivo da pesquisa. O questionário, que está apresentado no Apêndice A, foi composto por 23 questões, sendo 11 fechadas e 12 abertas. As questões fechadas foram elaboradas para obtenção de informações sociodemográficas dos profissionais, tais como a idade dos participantes, Estado de residência (Paraná ou Santa Catarina), tempo de formação, nível acadêmico, tempo de atuação clínica, área de atuação, faixa etária para quem presta atendimento e se estava ativo profissionalmente durante a realização da presente pesquisa. As questões abertas tinham o objetivo de direcionar o entendimento da implicação do vínculo terapêutico na prática clínica fonoaudiológica, na ótica dos fonoaudiólogos.

Para verificação da tangibilidade do questionário, antes do envio do instrumento para todos os profissionais inscritos do CRFa 3, foi feito um estudo piloto que contou com a participação de 12 fonoaudiólogos residentes no Paraná e em Santa Catarina, captados pela técnica da bola de neve. Essa técnica qualitativa propõe que, após a localização de algumas pessoas que correspondam ao perfil do estudo, elas sejam convidadas a recomendar outros sujeitos capazes de integrar a pesquisa e, assim, progressivamente^(13)^. Esses 12 profissionais responderam, avaliaram e sugeriram melhorias ao instrumento de coleta de dados, possibilitando às autoras observarem lacunas no mesmo, as quais foram revistas e adequadas, viabilizando a aplicação do instrumento a um maior número de participantes.

### Participantes

Responderam ao estudo 146 fonoaudiólogos clínicos, com registro ativo no Conselho de Fonoaudiologia da 3ª região (CRFa 3), abrangendo dois Estados do sul do Brasil: Paraná e Santa Catarina. A coleta de dados foi organizada a partir da inclusão das respostas de 96 profissionais fonoaudiólogos, que cumpriam os critérios de elegibilidade. Os participantes tiveram suas identidades preservadas, sendo codificados e reconhecidos, na pesquisa, por meio de números arábicos, que vão de 1 até 96.

Em função de critérios de inclusão, foram considerados profissionais com tempo de atuação clínica, igual ou maior de um ano. Por outro lado, tendo em vista os critérios de exclusão, foram retirados do estudo profissionais que não estavam atuando na clínica fonoaudiológica, no momento da coleta de dados.

### Procedimentos de coleta de dados

O instrumento eletrônico de coleta de dados foi encaminhado via Conselho Regional de Fonoaudiologia 3ª região (CRFa 3), pelo ambiente virtual *e-mail*, para 4.297 profissionais fonoaudiólogos, inscritos no órgão. Juntamente com o questionário eletrônico, foi enviado o Termo de Consentimento Livre e Esclarecido (TCLE), bem como um resumo do projeto, o qual explicava o seu objetivo e justificativa. No dia 13 de março de 2021, o questionário foi enviado aos profissionais e, devido ao baixo número de respostas, no dia 26 de abril o questionário foi reencaminhado, novamente, pelo CRFa 3. Assim, compuseram a pesquisa todos os profissionais que se mobilizaram para responder o questionário e que se adequaram aos critérios de inclusão, no período de dois meses, após o primeiro envio do instrumento.

### Análise dos dados

A análise dos dados apoiou-se na Análise de Conteúdo (AC), que apresenta caráter quanti-qualitativo, permitindo utilizar parâmetros estatísticos para estudar os fenômenos da comunicação^(14)^. Trata-se de um método de análise que considera aspectos objetivos e subjetivos de textos produzidos durante a pesquisa, a fim de discutir os dados coletados por meio de procedimentos sistemáticos capazes de organizar indicadores quantitativos e qualitativos^(15,16)^. De forma específica, a AC é definida como um conjunto de instrumentos metodológicos, que analisa diferentes fontes de conteúdo verbais e não-verbais, de forma refinada e crítica, aumentando o seu nível de efetividade^(16)^.

#### Análise quantitativa

A análise quantitativa buscou caracterizar o perfil sociodemográfico dos profissionais fonoaudiólogos participantes, através de análise estatística descritiva. Para verificar a existência de associação entre as diferentes variáveis categóricas avaliadas, foi utilizado o teste Qui-quadrado. Todas as análises foram realizadas no software estatístico Jasp, versão 0.14.1, sendo adotado um nível de significância de 5%.

#### Análise qualitativa

No que se refere aos materiais linguístico e discursivo coletados por meio da aplicação do questionário, que buscou apreender o que os profissionais fonoaudiólogos compreendem sobre o vínculo terapêutico no campo da Fonoaudiologia, o mesmo foi organizado seguindo os procedimentos da AC^(14)^. Nessa direção, para o tratamento dos dados, foram elaboradas análises temáticas e lexicais, as quais organizaram as categorias do presente estudo.

A organização ocorreu seguindo as três fases da AC: 1) a Pré-análise, em que se dá a preparação do material obtido na coleta de dados; 2) a Exploração do material, etapa em que as categorias são agrupadas a partir das unidades de registro, em função de suas características comuns, em torno dos vocábulos e dos temas referidos pelos participantes; 3) o Tratamento dos Resultados, fase em que se realiza a interpretação dos dados da pesquisa, sendo possível produzir inferências^(17)^.

## Resultados

A Tabela 1, apresentada a seguir, apresenta o perfil dos 96 profissionais participantes, considerando seus aspectos sociodemográficos.

**Tabela 1:** Características da população de estudo

*Variável*		*n*	*%*
Tempo de formação			
	*3-7 anos*	26	21,1
	*8-14 anos*	*19*	*19,8*
	*15-19 anos*	*15*	*15,6*
	*Acima de 20 anos*	*36*	*37,5*
Nível acadêmico			
	*Graduação*	18	18,8
	*Especialização/aperfeiçoamento*	58	60,4
	*Mestrado*	16	16,7
	*Doutorado*	4	4,2
Tempo de atuação clínica terapêutica			
	*1-4 anos*	15	15,6
	*5-9 anos*	*23*	*24*
	*10-19 anos*	*25*	*26*
	*Acima de 20 anos*	*33*	*34,4*
Área de atuação*			
	*Disfagia/Motricidade*	*66*	*68,8*
	*Linguagem*	*82*	*85,4*
	*Voz*	*27*	*28,1*
	*Audiologia*	*17*	*17,7*
Busca por alguma formação específica sobre o vínculo terapêutico			
	*Sim*	*29*	*30,2*
	*Não*	*67*	*69,8*
Tipo de formação especificamente voltada para ampliar compreensão sobre vínculo terapêutico. *			
	*Cursos*	*22*	*75,9*
	*Seminários*	*5*	*17,2*
	*Congressos científicos*	*2*	*6,9*
	*Palestras*	*15*	*51,7*
	*Grupos de estudos*	*16*	*55,2*
	*Outros*	*7*	*24,1*
Idade (anos) - Média (DP)		40.5 (11.2)	

* Havia a possibilidade de o profissional selecionar mais de uma resposta nessa questão

Fonte: Os Autores

Dos 96 fonoaudiólogos incluídos, 100% estavam ativos profissionalmente e atuando como fonoaudiólogos clínicos. No que concerne ao tempo de formação, 37,5% eram fonoaudiólogos formados a mais de 20 anos, seguido dos profissionais com três a sete anos de formação, perfazendo 21,1%. Em relação ao nível acadêmico, observou-se uma predominância de 60,4% de participantes com especialização/aperfeiçoamento, seguida de 18,8% que possuíam somente graduação, sendo que apenas 4,2% apresentavam doutorado.

Do total de profissionais que participaram deste estudo, 34,4% atuavam clinicamente a mais de 20 anos e 15,6% entre um e quatro anos. Com relação a área de atuação, há um número maior de fonoaudiólogos participantes que atuavam na área da linguagem, com 85,4%, seguido dos atuantes na área de disfagia/motricidade, representando 68,8% dos participantes. Com menor número representacional estão os profissionais da área da voz, com 28,1% e da audiologia, com 17,7%.

No que concerne a formação complementar voltada especificamente ao vínculo terapêutico, apenas 30,2% buscaram aprofundamento sobre a temática. E quando esses 30,2% foram questionados sobre o tipo de formação que realizaram, 72,9% afirmaram ter frequentado cursos, 55,2% participaram de grupos de estudos, 51,7% de palestras, 17,2% de seminários, 6,9% participaram de congressos científicos e 24,1% buscaram outros meios para aprofundar os seus conhecimentos sobre vínculo terapêutico.

### Eixo 1 - Representação do vínculo terapêutico

A Tabela 2, apresentada a seguir, busca explicitar a compreensão que os participantes da pesquisa têm acerca do vínculo terapêutico no contexto clínico fonoaudiológico. Para tanto, os participantes responderam o que é vínculo na sua visão. E a explicitação das respostas dadas pelos fonoaudiólogos foi organizada nas categorias: relação/interação, confiança/acolhimento, escuta/empatia e outros.

**Tabela 2:** Caracterização das respostas obtidas sobre vínculo terapêutico

**EIXO 1 - REPRESENTAÇÃO DO VÍNCULO TERAPÊUTICO**			
Explicitação do que é vínculo terapêutico na visão dos fonoaudiólogos			
Categorias	*n*	*%*	Exemplos de unidades de registro
*Relação/interação*	46	47,9	A estreita relação/interação entre paciente/terapeuta (1)
			Interação entre profissional, paciente e família (43)
*Confiança/acolhimento*	*25*	26	Confiança da família nas decisões do terapeuta (22)
			O Vínculo terapêutico passa, acima de tudo, por confiança entre paciente e terapeuta e acolhimento ao paciente (8)
*Escuta/empatia*	*2*	2,1	Um processo que se dá/acontece a partir da escuta qualificada e ativa do terapeuta (14)
			Tem a ver com empatia (47)
*Outros*	*15*	15,6	Atrelar o atendimento com equipe multidisciplinar (10)
			Incluir a família no processo terapêutico (60)
*Sem resposta*	*8*	8,3	

Fonte: Os Autores

No eixo 1, a maior parte dos profissionais definiu vínculo terapêutico como sendo *relação/interação,* representando 47,9% do total de participantes. Outros 26% o definiram como *acolhimento/confiança.* Entretanto, chama atenção o fato de 26% não responderem à questão.

### Eixo 2 - O papel do vínculo terapêutico na visão de fonoaudiólogos

Para organização desse eixo, os profissionais foram questionados sobre qual o papel do vínculo terapêutico para a prática clínica fonoaudiológica. E as respostas foram agrupadas em categorias relacionadas a afinidade/confiança, base/fundamental, motivação/engajamento, evolução/desenvolvimento e outros, conforme ilustrado pela tabela 3.

**Tabela 3:** Caracterização das respostas obtidas sobre o papel vínculo terapêutico

**EIXO 2 - O PAPEL DO VÍNCULO TERAPÊUTICO NA VISÃO DE FONOAUDIÓLOGOS**			
Categorias	*n*	*%*	Exemplos de unidades de registro
*Afinidade/confiança*	12	12,5	Só é possível realizar um trabalho terapêutico mediante confiança que se estabelece no vínculo (50)
			Sem o vínculo não há troca na relação e não há confiança do paciente e/ou familiar (9)
*Base/fundamental*	37	38,5	Papel fundamental, pois sem vínculo dificilmente conseguiremos alcançar nossos objetivos (17)
			Acredito ser um dos pilares necessários para o sucesso terapêutico (21)
*Motivação/engajamento*	8	8,3	É o que motiva (4)
			É o primeiro passo facilitador do engajamento terapêutico (55)
*Evolução/desenvolvimento*	24	25	Ampliar possibilidades do desenvolvimento do paciente (7)
			É necessário para evolução do paciente (45)
*Outros*	*12*	12,5	Sem vínculo terapêutico cancelo tratamento (63)
			Fortalece as estratégias planejadas (87)
*Sem resposta*	*3*	3,1	

Fonte: Os Autores

Referente ao papel do vínculo terapêutico para o trabalho clínico fonoaudiológico, os profissionais o descreveram, de forma mais significativa, como sendo a *base/fundamental*, representando 38,5% das respostas, e necessário para a *evolução/desenvolvimento do paciente*, de acordo com 25% das respostas, 8,3% relacionaram vínculo à *motivação/engajamento no trabalho terapêutico*, 12,5% descreveram outras características e 3,1% não responderam à pergunta.

### Resultados quantitativos

Houve associação entre o tempo de formação dos participantes e as respostas desses participantes com relação ao papel vínculo terapêutico (p = 0.036). Para os profissionais que concluíram a graduação há mais de 20 anos, o vínculo é entendido como fundamento da clínica fonoaudiológica. E para os profissionais que concluíram a graduação há menos de 14 anos e há mais de oito anos, o vínculo é tomado como responsável pela evolução e pelo desenvolvimento do trabalho terapêutico (Figura 1A).

**Figura 1:** Demonstração gráficas dos resultados dos cruzamentos das informações que apresentaram significância

Legenda: Teste qui-quadrado; Nível de significância 5%

Verificou-se, também, associação entre o tempo de atuação clínica e as respostas dos participantes quanto ao papel do vínculo terapêutico (p= 0.050). Para os fonoaudiólogos que atuam, clinicamente, há mais de 20 anos, o vínculo terapêutico foi postulado como a base de seu trabalho. E, para os profissionais que atuam há mais de cinco e há menos de 10 anos, o vínculo é apontado como elemento que leva o trabalho terapêutico a se desenvolver (Figura 1B).

## DISCUSSÃO

Observou-se, nos resultados explicitados na Tabela 1, quanto à caracterização dos participantes, que parcela significativa dos profissionais são formados e atuam clinicamente, como fonoaudiólogos, há mais de 20 anos, evidenciando um interesse maior destes em colaborar com a presente pesquisa, que se volta à temática do vínculo terapêutico na clínica fonoaudiológica. Esse dado indica que o papel do vínculo tende a se tornar mais significativo na medida em que os profissionais têm mais tempo de formação e, portanto, de atuação clínica.

Em relação a área de atuação dos participantes, chama atenção a predominância de profissionais que atuam na área da linguagem. A predominância de fonoaudiólogos que atuam clinicamente na área da linguagem pode ser justificada pelo fato de a linguagem ser um dos pilares essenciais para o estabelecimento e sustentação do vínculo. Pois, é por meio da linguagem que todo ser humano se constitui como sujeito, compreende o mundo e age sobre ele^(18)^. O vínculo terapêutico permite, por via da linguagem, acessar os conteúdos conscientes e inconscientes do sujeito, trazendo à tona a sua subjetividade, tão necessária para o encaminhamento do trabalho clínico fonoaudiológico^(19)^.

Além disso, o vínculo terapêutico é associado a significativas melhorias das habilidades comunicativas de sujeitos em tratamento fonoaudiológico^(2)^. A relação terapêutica deve, também, ser considerada na reabilitação de sujeitos que apresentam dor orofacial crônica. Segundo a autora, tal relação amplia a escuta do terapeuta, sendo possível auxiliar o sujeito no alívio de sua dor, na medida em que a sua história de vida é considerada para a compreensão de seus sintomas^(4)^.

Convém ressaltar que, apesar de a literatura da área da audiologia reconhecer a importância do vínculo entre o fonoaudiólogo e o paciente no trabalho clínico audiológico, foi restrito o número de audiologistas que aceitaram participar da presente pesquisa. De acordo com estudo voltado à reabilitação audiológica de pessoas idosas, o vínculo terapêutico é considerado fundamental, na medida em que impacta na diminuição do adiamento de consultas e na adesão ao tratamento proposto^(20)^. Nesse mesmo estudo, o vínculo, também, está associado ao fato dos pacientes se sentirem valorizados e escutados pelo profissional e não apenas concebidos como consumidores e compradores de próteses auditivas^(20)^.

Outro ponto que merece atenção, nos resultados evidenciados na Tabela 1, é que praticamente 70% dos profissionais não fizeram capacitações específicas sobre temáticas que envolvem o vínculo terapêutico, para que pudessem fundamentar a sua prática clínica. Portanto, embora os profissionais apontem o vínculo terapêutico como um pré-requisito essencial para o encaminhamento de seu trabalho, eles não estão sendo capacitados para dar conta desse pré-requisito fundamental relacionado ao seu trabalho. É preciso refletir acerca dos motivos capazes de explicar essa situação, que podem estar relacionados a falta de interesse ou, mais do que isso, a falta de oferta de ações formativas para suprir tal demanda.

### Eixo 1 - Representação do vínculo terapêutico

No eixo 1, no qual, de acordo com a Tabela 2, está explicitado o que os fonoaudiólogos entendem por vínculo terapêutico, indica uma predominância significativa de profissionais que o compreendem como a relação que se estabelece entre os participantes do processo terapêutico, o fonoaudiólogo e o paciente, sendo essa relação a linha condutora, responsável pelo encaminhamento do trabalho fonoaudiológico. Sobre essa questão, de acordo com estudos psicanalíticos, se faz necessário compreender que o vínculo terapêutico é uma atividade dinâmica, em constante movimento, que compreende tanto os sujeitos envolvidos, quanto as suas histórias pregressas^(21)^.

Nessa direção, a relação vincular, constituída em âmbito terapêutico, é perpassada pela coexistência do paralelo entre as experiências afetivas vivenciadas pelos sujeitos ao longo de sua história e as experiências relacionais elaboradas no espaço terapêutico, conforme proposto pela teoria do apego^(6)^. Também há a necessidade de o paciente ser considerado, compreendendo-o como um ser biopsicossocial, para que seja possível ressignificar a angústia e o sintoma que se apresenta a ele. E tal feito só se torna possível por meio da relação estabelecida entre paciente e terapeuta, a qual permite ao profissional reconhecer a necessidade do sujeito que o busca e, assim, intervir de forma mais efetiva, auxiliando-o na mitigação de seu sofrimento^(22,23)^.

Nessa perspectiva, seguindo com o primeiro subeixo, outra parte dos participantes da pesquisa relacionaram vínculo terapêutico à escuta, confiança, acolhimento, entre outros. Uma escuta terapêutica qualificada promove uma atenção holística, capaz de fortalecer laços vinculares, viabilizando uma aproximação entre o profissional e as verdades fidedignamente relacionadas ao real sofrimento do sujeito^(24)^. De acordo com estudos da Saúde Coletiva, uma escuta qualificada maximiza o potencial terapêutico e contribui para a consolidação da relação terapêutica, na medida em que respeita a singularidade do paciente, fortalecendo a confiança e a participação dos envolvidos no processo terapêutico^(7)^.

No que concerne a confiança, a mesma deve ser considerada como um dos pontos primordiais do trabalho terapêutico, pois permite que o paciente se sinta à vontade e seguro para compartilhar a sua história de vida, promovendo interações mais autênticas, rumo a reelaboração do sintoma. Mas, tal confiança só se torna possível com base no acolhimento direcionado ao paciente, levando- o a se sentir seguro para trazer à tona a sua singularidade^(25)^.

### Eixo 2 - O papel do vínculo terapêutico na visão de fonoaudiólogos

Acerca do papel do vínculo terapêutico, na prática clínica fonoaudiológica, observou-se que 38,5% dos profissionais entende o vínculo terapêutico como base fundamental para sua prática clínica. O vínculo terapêutico é apontado como primordial para o delineamento do trabalho terapêutico, pois, para além da construção de uma base segura que auxilia o paciente a abordar as suas demandas, a relação entre o próprio paciente e o clínico pode contribuir para que o sujeito, em sofrimento, possa ressignificar os seus vínculos afetivos anteriores. Conforme a teoria do apego, principalmente, em circunstâncias nas quais os sujeitos cresceram em contextos interpessoais baseados na desconfiança e no medo, o terapeuta pode assumir uma posição capaz de promover um vínculo seguro que leve o paciente a se deparar com um outro modelo de relação, apoiado em um elo que viabilize confiança e segurança, reelaborando relações difíceis vivenciadas em situações pregressas^(3)^.

Também, segundo uma perspectiva psicanalítica, o vínculo terapêutico deve ser considerado como fator subjacente a todo fazer clínico. Pois, é uma condição necessária para o encaminhamento do trabalho terapêutico, ou seja, um elemento que perpassa toda a atividade clínica, sem o qual, tendenciosamente, não há possibilidades de tratamento, cuidado e/ou ressignificação do sintoma^(19)^.

Motivação e engajamento foram associados ao papel do vínculo terapêutico, na visão de 8,3% dos fonoaudiólogos, participantes da presente pesquisa. Segundo estudo pautado na saúde coletiva, a relação terapêutica, na medida em que abre espaço para que o sujeito explicite as suas particularidades, permite que ele compreenda o seu papel no trabalho a ser desenvolvido, levando-o a assumir uma posição ativa na relação com o clínico^(26)^. Nessa direção, o vínculo terapêutico é capaz de potencializar, de forma significativa, o engajamento, a motivação, a autonomia e participação social dos sujeitos dentro e fora do *setting* terapêutico^(9)^.

Parte considerável de 25% dos fonoaudiólogos relacionou o vínculo à evolução e ao desenvolvimento da terapia. Sobre essa relação, cabe ressaltar que o vínculo terapêutico não deve ser associado apenas a “cura” ou supressão do sintoma, mas, como uma oportunidade que permite ao paciente ressignificar a sua história de vida, suas dores e vínculos anteriores. De acordo com a Política Nacional de Promoção da Saúde, os cuidados voltados a saúde não deveriam se limitar somente a recuperação da doença, mas a uma atividade na qual o profissional, mediante a uma escuta qualificada e acolhedora, promova a maximização da autonomia, empoderamento e participação social do paciente, auxiliando-o no processo de se tornar autor da sua própria história de forma ativa e consciente^(27)^.

## CONSIDERAÇÕES FINAIS

No que se refere especificamente a compreensão dos profissionais fonoaudiólogos acerca dos sentidos que o vínculo terapêutico assume na prática clínica fonoaudiológica, o mesmo foi postulado como parte essencial para o delineamento e desfecho do processo terapêutico, sendo capaz de potencializar os fatores de motivação, engajamento e ajuda ao sujeito, na ressignificação de seu sintoma.

## Apêndice A Questionário estruturado para coleta de dados

**Table:**

DADOS DE CARACTERIZAÇÃO:
1. Idade: _________
2. Estado em que reside:
() Paraná () Santa Catarina
3. Trabalha em:
() Capital () Região Metropolitana () Interior
4. Tempo de formação na graduação:
a) () 1 a 2 anos e 11 meses
b) () 3 a 7 anos e 11 meses
c) () 8 anos a 14 anos e 11 meses
d) () 15 anos a 19 e 11 meses
e) () acima de 20 anos
5. Instituição onde se graduou?
6. Nível acadêmico:
a) () graduação
b) () Especialização Em qual área?
c) () Mestrado Em qual área?
d) () Doutorado Em qual área?
7. Está profissionalmente ativo atualmente?
a) () Sim
b) () Não
8. Área(s) de atuação: * Lembrando que pode haver mais de uma resposta
a) () Linguagem, há quanto tempo?
b) () Disfagia, há quanto tempo?
c) () Motricidade, há quanto tempo?
d) () Fonoaudiologia Educacional, há quanto tempo?
e) () Voz, há quanto tempo?
f) () Gerontologia, há quanto tempo?
g)() Audiologia, há quanto tempo?
h)() Fonoaudiologia Neurofuncional, há quanto tempo?
i) () Saúde Coletiva, há quanto tempo?
j) () Fonoaudiologia do Trabalho, há quanto tempo?
h)() Neuropsicologia, há quanto tempo?
l) () Fluência, há quanto tempo?
9. Qual seu campo de atuação: * Lembrando que pode haver mais de uma resposta
a) () Clinica
b) () Hospitais/maternidades
c) () Home Care
d) () Unidade de saúde
e) () Instituições de ensino superior
f) () Empresas/Industrias
g) () Escolas regulares/Especial
h) () Outros, Quais?
10. Você realiza atendimento clínico terapêutico há quanto tempo?
a) () menos de um ano
b) () 1 a 4 anos e 11 meses
c) () 5 anos a 9 anos e 11 meses
d) () 10 anos a 19 e 11 meses
e) () acima de 20 anos
11. Qual faixa etária(s) você presta atendimento? * Lembrando que pode haver mais de uma resposta.
a) () Bebês - de 0 a 1 ano
b) () Crianças - de 2 a 11 anos
c) () Adolescentes - de 12 a 18 anos
d) () Adultos
e) () Idosos
Questões relacionadas ao vínculo terapêutico na prática clínica fonoaudiológica:
12.O que você entende por vínculo terapêutico?
13.Se você pudesse explicitar vínculo terapêutico em uma palavra, qual palavra usaria?
14. Você busca estabelecer vínculo terapêutico com as pessoas que atende clinicamente?
a) () Sim
b) () Não
Justifique:
15. Como você percebe que o vínculo foi estabelecido?
16. Qual é a relevância do vínculo no decorrer do processo terapêutico?
17. Qual é o papel do fonoaudiólogo no estabelecimento do vínculo terapêutico?
18. Você já se viu diante de casos em que foi difícil estabelecer vínculo terapêutico?
a) () sim
b) () não
Em caso positivo, na sua opinião, que fatores estiveram envolvidos nessa dificuldade?
19.Se, na clínica, você busca estabelecer vínculo, tal estabelecimento está pautado(a) em alguma abordagem teórica?
a) () Sim
b) () Não
Se sim, qual?
20.Você buscou alguma formação mais específica sobre vínculo terapêutico?
a) () Sim
b) () Não
Se sim, que tipo de formação?
a) () cursos
b) () palestras
c) () seminários
d) () congressos científicos
e) () grupos de estudos
f) () outros: quais ________________________________________________
21. Outras observações/ comentários/depoimento que queira fazer:
